# Implementation of Knowledge Management in Higher Education: A Comparative Study of Private and Government Universities in India and Abroad

**DOI:** 10.3389/fpsyg.2022.944153

**Published:** 2022-06-16

**Authors:** Dhruv Galgotia, Nirupa Lakshmi

**Affiliations:** ^1^Department of Management Studies, Dr. A. P. J. Abdul Kalam Technical University, Lucknow, Uttar Pradesh, India; ^2^Department of Management Studies, Galgotias College of Engineering and Technology, Uttar Pradesh, India

**Keywords:** high education, knowledge management, private, public, universities

## Abstract

All academic institution contributes to the corpus of knowledge in some way. To keep expanding, the resulting information and data must be collected in a single area and shared throughout society. Knowledge developed in academic institutions is not adequately preserved or gathered, according to research. It has also been observed that the majority of the content or knowledge developed in academic institutes is unknown to the general public and is categorized as a literature review, which may be useful if proper coding is kept in the organization. The purposeful integration of humans, processes, and technology dedicated to developing, capturing, and executing an organization’s creative infrastructure is known as a Knowledge Management (KM) method. Knowledge Management supports educational institutes in refining their capability to acquire and share information and knowledge, applying it to problem resolution and promoting research and continuous development. This paper advocates a holistic strategy for finding, analyzing, recording, retrieving, and distributing the whole of an administration’s data properties. Databases, records, procedures, regulations and hitherto un-captured knowledge and talent in ordinary employees are samples of these properties. Higher education institutions can use KM as a tried and true approach to dealing with their problems. KM aids in the motivation of research as well as the promotion of partnerships and innovations in the future.

## Introduction

The purposeful integration of humans, processes, and technology dedicated to developing, capturing, and executing an organization’s intellectual infrastructure is known as a knowledge management method. It allows employees inside a company to communicate what they know, resultant in better amenities and outcomes (Ramachandran et al., 2009). Through the exchange of best practices, good decision making, quicker reaction to significant institutional challenges, proper process handling, and increased people skills, KM plays a vital role in improving high organizational performance. As a result, there will be less need to recreate the wheel, more targeted and relevant policies in line with institutional aims and objectives, faster access to information, enhanced administrative and academic services, lower costs, and fewer errors and failures. Unfortunately, few Higher education institutions (HEIs), if any, accomplish all or even the majority of these rewards in practice (Mazhar and Akhtar, 2016). The success of KM projects appears to be attributable to a lack of distribution culture, a lack of understanding of the assistance of KM, and an inability to incorporate KM into daily working practices. The massive development in the population of higher institutes in India over the last era has put tremendous pressure on schools to perform better under intense competition (Bhusry et al., 2011).

Higher education institutions are made up of a variety of administrative and academic procedures that generate knowledge as part of their operations. The challenge is whether the appropriate use of this knowledge asset adds value to the products and services they provide. HEIs must align themselves in order to establish strategies for using institutional knowledge to improve their efficiency of operations. This necessitates quick responses to rapidly changing technology and rising academic needs (Galgotia, n.d.). To do so, the organization’s knowledge must be properly discovered, captured, transformed, and communicated. This opens the door for organizations to understand the critical need for organizational learning. The use of an understanding management approach will mean reinforcing to obtain a comprehensive, reactionary, and integrative perspective of information within the organization for use in merge issues, resulting in improved knowledge distribution and further planning, decision making, and quality enhancement (Nawaz, 2015).

Individuals, groups, and entire organizations may jointly and systematically develop, share, and utilize information to better achieve their goals through knowledge management. KM fosters exceptional cooperation in order to maximize the value of the organization’s knowledge and information assets, subsequent to augmented efficiency and creativity. “Knowledge” is defined as “the insights, interpretations, and practical know-how that we all have.” It distinguished between two categories of knowledge: explicit and inferred knowledge (Sardjono and Firdaus, 2020). Tacit knowledge is information that is comprehended and implemented without conscious thought. Tacit knowledge is highly customized, based on experience and impacted by the individuals’ views, viewpoints, and values. It is tough to codify and only exists in the imaginations of those who have it. It is often communicated through highly engaging dialog and shared memories. On the other hand, knowledge acquisition is simple to explain, capture, and communicate in many ways. It is serious and methodical (Yeh, 2005).

**Figure 1:**
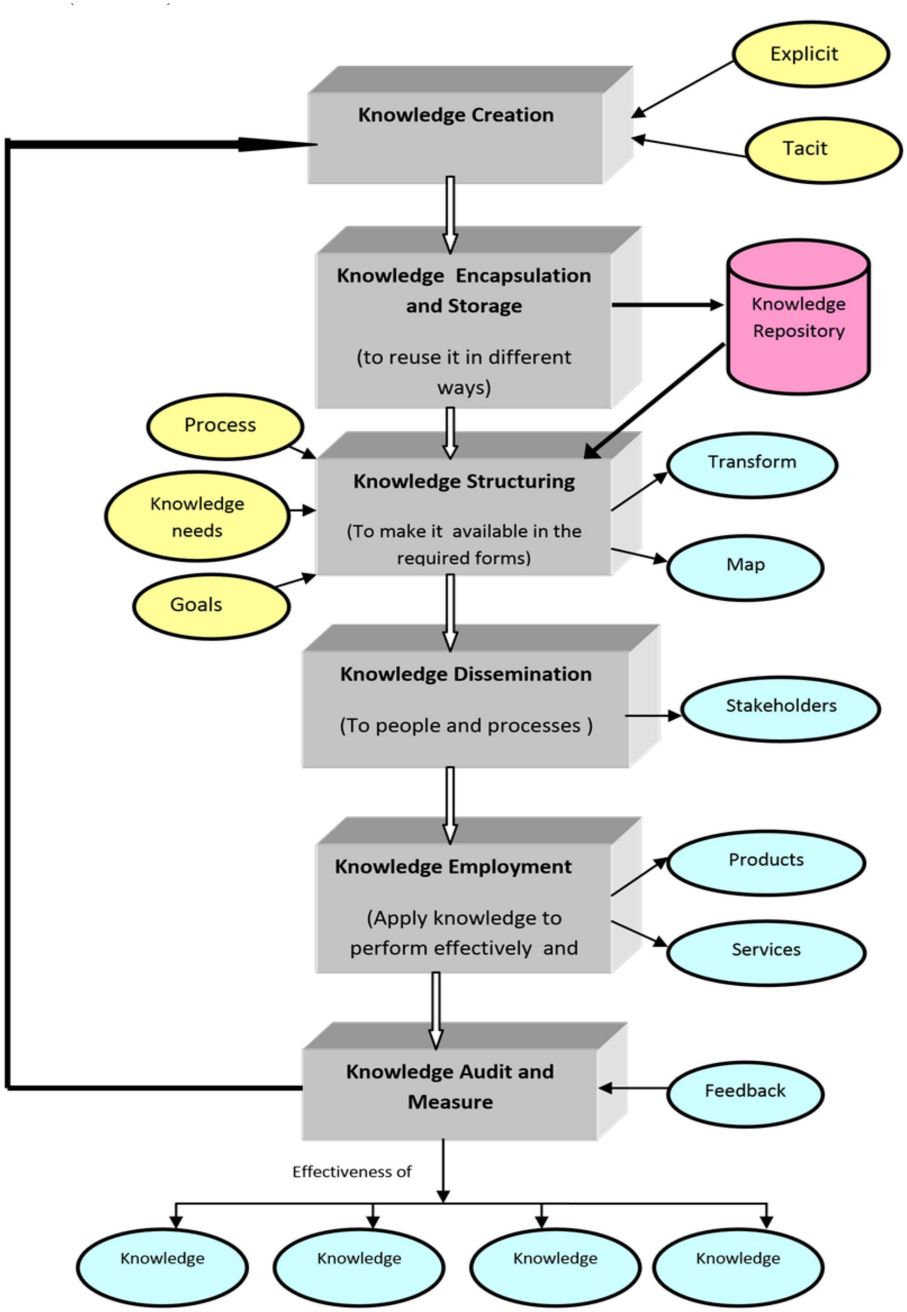
This will show the Knowledge Management Structure for understanding the process which performs in Knowledge Management (Escorcia Guzmán and Barros Arrieta, 2020).

The approach includes evaluating the current gap in the group’s knowledge needs and proposing an iterative strategy for bridging the gap. It aims at identifying higher education institutions’ strategic needs depending on organizational objectives, organizational hierarchical system, stakeholders, and procedures (Horban, 2021). The next stage is to identify the knowledge fissure as well as the variables that contribute to it (Nur et al., 2017). Figure 1 depicts the requirement to reduce the gap for effective utilization of organizational knowledge against aims and priorities.

Explicit knowledge may be easily recorded and shared. This information is more easily shared and utilized within the company. Organizations use knowledge management systems to achieve their goals of enhanced performance, expertise transfer, competitive advantage, and the growth of collaborative practices (Alksasbeh et al., 2018). KM is defined as the “identifying, expansion, and successful use of essential knowledge in an organization.” Knowledge management is defined as “a methodical, comprehensive approach to improving the long-term knowledge management at all levels of the company.” Knowledge management, as according, is the act of finding, expanding, and successfully utilizing an organization’s current knowledge to fulfill the organization’s objectives while also fostering an organizational culture that encourages knowledge development (Sirajuddin et al., 2005). These and many other perspectives on knowledge management suggest that an effective knowledge management system should be incorporated into people’s everyday routines, allowing for a continual flow of information across the company. Capturing, storing, generating, and sharing organizational information is the foundation of a knowledge management system. IT is a critical facilitator for KM system, allowing for the capture, transformation, storage and distribution of acquaintance (Muftahu and Jamil, 2021).

### The KM in Higher Education in India

During the administrative and academic procedures of higher education institutions, knowledge is created. Individuals develop knowledge in the form of explicit knowledge in the form of papers, methods, and outcomes, and tacit information in the procedure of experience, judgments, perspectives, and discernments (Hoffmann et al., 2019). The problem is figuring out how to make this tacit and explicit information available to the institution as a centralized resource. The ability to capture and make available institutional memory will maintain continuity and expedite institutional learning. Instead, most HEIs are faced with the arduous issue of integrating their institutional memory in order to promote information exchange and judgment. Knowledge is developed in a variability of ways at various levels, and it is compulsory in a diversity of ways at each level.

Teaching, assessment, evaluation, counseling, admissions, training, placement, and development and research are all administrative and academic activities that result in many important practices and studies that may be regarded as information in the framework of higher educational establishments (Savitri et al., 2013; Acevedo-Correa et al., 2020). Faculty, administration, academics, students, research, training and placement are only a few of the components or levels that make up an academic institution. All of these levels both develop and consume information, albeit the kind of knowledge differs depending on the level. It is critical to determine the information so each layer gives to the system and the information that each level needs to accomplish its duties and to discover effective ways to apply this knowledge at the locations of usage (Tejedor et al., 2019). The information demands at all stages must be met by a comprehensive KM system.

In the academic world, knowledge management is a relatively recent discipline. Many future national and international events and seminars will focus on knowledge management (Prahiawan et al., 2021). Many international universities are actively involved in knowledge management operations and research (Masete and Mafini, 2018). It is now gaining popularity in the sphere of education because of the necessity to reveal the intellectual potential accessible in institutions for the purpose of exchanging experiences. It has a lot of promise and should be just as important, if not more so, in the education sector. Information builds on previous knowledge, and historical events aid in the generation of new knowledge (Rahman Ahmad et al., 2020). Human efforts, which are produced *via* effective educational activities, scientific research, and producing novel concepts in the area of interest, are the primary source of knowledge development. All experience and understanding institutions, such as companies, Research and Development (R&D) centers, and higher ed. academics from colleges to universities, are on the lookout for new ideas in their fields of study and contribute to the understanding in a variety of ways.

Management theorists and practitioners like Peter Drucker and Paul Strass Man wrote paper in the 1970s that gave birth to knowledge management. These publications look at how knowledge and information may be used as important organizational resources. Knowledge management (KM) plays dynamic role which is critical in higher education institutions (HEIs), notably in terms of planning, organizing, monitoring, and managing KM assets connected to intellectual capital (de la Torre et al., 2021; Horban, 2021; Migdadi, 2021; Pereira et al., 2021; Terán-Bustamante et al., 2021). As a result, knowledge management might improve knowledge exchange and overall performance. Knowledge management enables for improved creativity inside the firm, increased access to best practices for consumers, and lower staff turnover. Every year, the importance of knowledge management grows rapidly in various places such as universities and organization all around the world.

Higher education may use knowledge management to improve efficiency, productivity, cooperation, and employee happiness while also encouraging high-level planning and innovation. According to a study, knowledge management created in academic institutions is not sufficiently maintained or gathered (Bratianu et al., 2021; Indrašienė et al., 2021; Sekli and De La Vega, 2021). The majority of the information or knowledge created in academic institutes is unknown to the general public and is classified as a literature review, which may be valuable provided correct coding is maintained in the organization.

### Proposed Solution

Knowledge Management assists educational institutions in improving their capacity to acquire and share information and knowledge, apply to resolve many issues, promotes research and continuous development in the field of knowledge (Purwanto et al., 2020). Traditional knowledge management systems are focused on the ability to capture knowledge in centralized systems. Knowledge management builds upon collegial and professional teamwork by actively engaging people in sharing with others what they know and what they are learning (Ammirato et al., 2021; González-Campo et al., 2021). This paper provides a comprehensive approach to locating, analyzing, recording, retrieving, and disseminating an administration’s data attributes which help to understand the role of the Knowledge Management in private and government universities and how it will provide effective outcomes for the educational system.

## Literature Review

Higher education is significantly involved in knowledge-related activities, but they are also accountable for knowledge generation, interchange, preservation, distribution, re-use, and learning. In higher education, KM approaches can improve administrative and academic services while also lowering expenses. Colleges and universities have a significant chance to use KM strategies to support all aspects of their assignments, including instruction, social utility, and research. The development, enhancement, preservation, and protection of knowledge are all aspects of knowledge management in higher education institutions. These institutions have immense potential and the capacity to build their own knowledge management since they were pioneers in the creation and distribution of information. The major engine that enhances the competitiveness, excellence, importance, and attractiveness of any higher education institution is the delivery of decent education and services related. The purpose of this work is to conduct an analysis of relevant literature on information management and its applicability to institutions of higher learning in particular.

Higher educational institutions (HEIs) develop and utilize knowledge. The expansion in the numbers of higher education institutions in India over the last period has boosted rivalry and pressure to perform better. As a result, institutions have been obliged to acknowledge the importance of knowledge management (KM) projects as a critical asset. The objective of this paper is to highlight the importance of KM in higher education institutions and to investigate the impact of an IT-based knowledge management intervention. The research investigates the many functions in higher education institutions, as well as the needles that influence these provinces. The authors assessed the functional provinces for IT-based knowledge management interventions and determined the apparent advantages. To support their findings, the authors provided conceptual frameworks for the effective collection, structuring, distribution, encapsulation and application of organizational knowledge more towards the aims and purposes of the organization. The authors believe that implementing the framework will improve the transition of organizational knowledge into judgment and actions (Bhusry et al., 2011).

Mazhar and Akhtar (2016) explained about the goal of this research work is to investigate (KM) methods at higher education institutions in Europe, Asia, and the Gulf Cooperation Council (GCC) countries, as reported by faculty members. Knowledge Perception (KP), Knowledge Creation (KC), Knowledge Sharing (KS), Knowledge Gathering (KG), Knowledge Diffusion (KD), and Knowledge Retention (KR) were analyzed and compared across the three areas to see how similar and different KM practices were. A systematic questionnaire was used to gather data, which was then sent online to faculty members from various institutions in Europe, Asia, and the Gulf Cooperation Council (GCC) countries. Scales for reliability, one-way ANOVA, and the *t*-test were used to evaluate, compare, and interpret the results in order to make relevant judgments. The findings show that there are no significant differences in KP, KC, KS, and KD amongst higher education institutions. However, there is a substantial difference between KG and KR. The study’s findings may be used to help higher education institutions from three distinct areas create best practices in knowledge management to improve performance (Mazhar and Akhtar, 2016).

## Methodology

The knowledge management practices of instructors at public and private institutions in India and overseas were investigated using a quantitative research technique and a cross-sectional design of the study.

### Design

A survey was done as part of the descriptive research. All HEC-recognized public and private universities in India and overseas were included in the study’s population. The sample was chosen using a multistage stratified random sampling procedure. At the outset, 10 universities from the public and private sectors were chosen at random. In the second step, faculties were randomly chosen from those institutions, and their methodologies for KM will be understood properly. Departments were chosen at random in the third step, and instructors were chosen at random in the final stage. The researcher individually visited each university to administer the device and collect the data from the university.

### Instrument

The authors used an interviewing and group debate study, as well as relevant expertise in higher educational institutions, to identify the functional areas in HEIs and the variables that promote the efficiency of KM in these areas. Involvements were also obtained from previous work in the field of knowledge management in higher education.

### Sample

The mean, variance, and percentage were calculated using descriptive statistical techniques. The *t*-test was used as an inferential analysis approach to compare the differences between public and private institutions. Collect the data from the different private and public universities of India and abroad. Here the authors considered 10 public and 10 private universities. The information gathered during groups and personal interviews with academics, departmental heads, deans, and employees, as well as observation of practices and methods, was used to define the areas in HEIs as well as the criteria that identify the domains. The information gathered was examined using the contents analysis method. Content analysis is examining the content of documentary sources such as books, periodicals, and newspapers, as well as oral materials such as interviews and focus groups, in order to identify particular qualities that may be quantified or tallied.

### Data Collection

**Figure 2:**
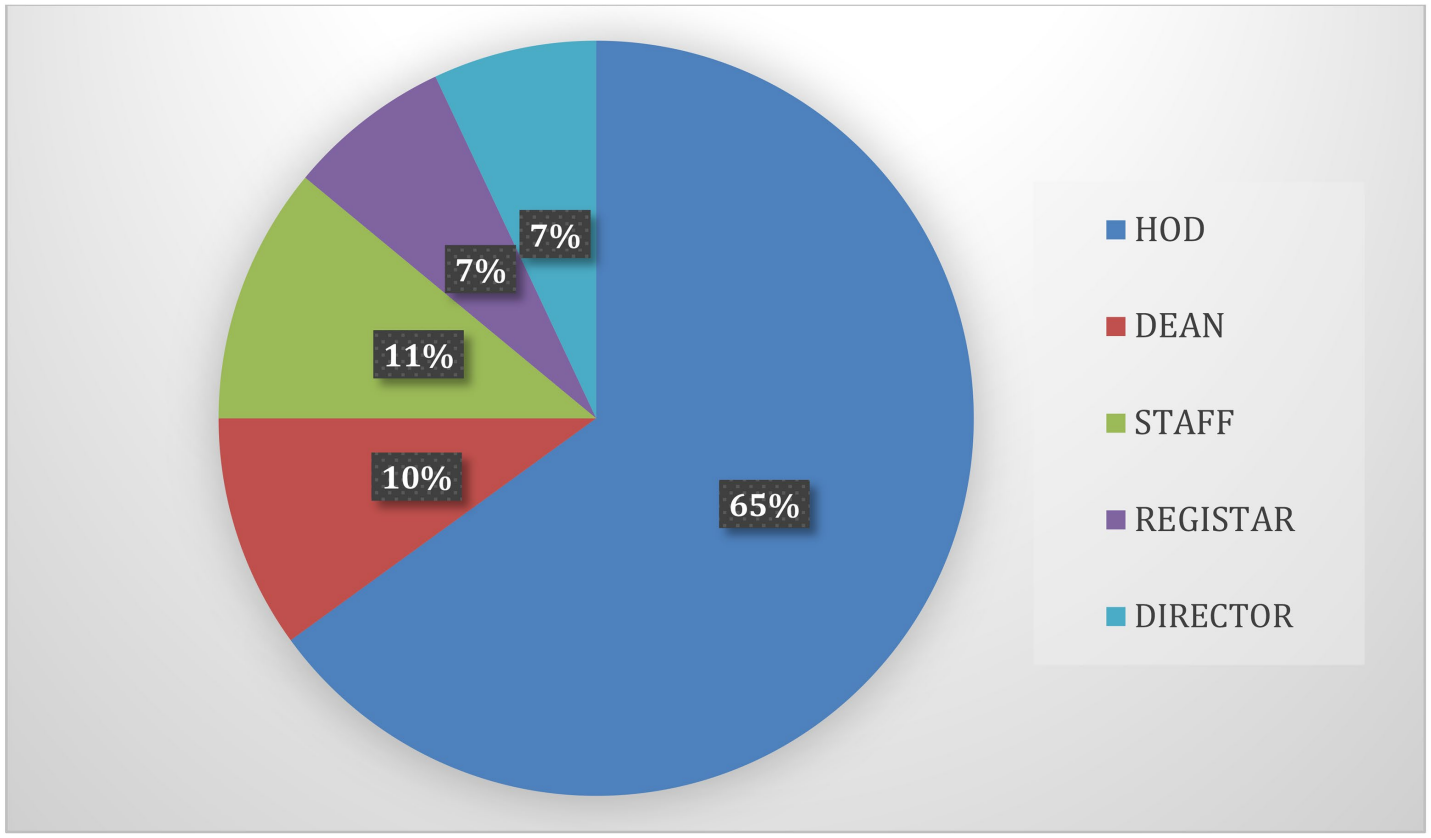
This graph shows the respondent’s role in the institution.

Areas and determinants are identified. The authors used an interviewing and group conversation-based study, as well as relevant expertise in educational institutions, to identify the domains in HEIs and the variables that promote the efficiency of KM in these areas. Inputs were also obtained from previous work in the field of knowledge management in higher learning. The gathered information through group and personal interviews with academics, heads of departments, deans, and employees, as well as inspections of practices and methods, was used to define the functional domains in HEIs and the criteria that identify the domains. The respondent role of the institute is shown in Figure 2.

### Data Analysis

The content analysis revealed the activity domains in higher education institutions and the factors that influence KM involvement in these areas. Institutional management and building, research, administrative support, purchase and sourcing, accounting, education—learning process, awareness process, admission, locations and faculty recruiting and selection, faculty performance appraisal, dean of students, and other domains were identified as the major realms. The authors limited their research to a few select domains which are shown in Table 1.

**Table 1:** This will show the author and the year in which they explain the knowledge management after analysis through their techniques.

Author & Year	Title	Objectives of the paper	Sector	Variable	Techniques	Results	Research gap
Ali et al., 2018	Gamification, serious games, simulations, and immersive learning environments in knowledge management initiatives	The goal of this study is to present the in and critical overview of the research and ideas on knowledge, knowledge management (KM), organizational learning, and the knowledge-based economy.	Education	Game-based learning	Various popular refereed journals concentrating on KM and gamification, as well as books, internet databases, governmental papers, and statistics, have all been studied and reviewed.	In the corporate world, game-based learning (GBL) must be aligned with the learning goals and outcomes of training and development, and it must clearly illustrate that learning can be assessed and attained. Through participant commitments, serious gaming experiences encourage human growth and transformation by instilling an attitude of acceptance of the challenge, determination to succeed, and ongoing creativity. Simulations force the participant to immerse themselves in a virtual world. Finally, gamification may be used as a wrapper for GBL, serious games, and simulations, or it can be used to educational and workplace activities as a set of strategies.	In higher education, GBL must be aligned with a program’s learning goals or outcomes, and it must clearly illustrate that learning can be assessed and completed through practical, project-based, competence-based learning methodologies.
Corcoran and Duane, 2018	A confirmatory factor analysis of knowledge management assessment instrument in Indian higher educational institutions	To explore the focus of KM research in HEIs in the region	Education	Enterprise Social Network Enablers	The research is structured as an Action Research project that spans three cycles over the course of a year. A conceptual model was constructed for empirical testing during the Diagnosing phase. 30 semi-structured interviews and a number of focus groups were used to gather data. Content analysis and reflective journaling were used to augment this.	The findings back up the conceptual framework by revealing the preconditions for establishing an organization social network-enabled knowledge-sharing environment, as well as the motivators and barriers to participation, as well as the perception organizational and individual advantages of increased employee experience and understanding activity.	To effectively manage the creation and evolution of the information sharing environment, it is also vital to understand both what inspires and stops the rest of the staff population from participating.
Baptista Nunes et al., 2017	Knowledge Management Practices in Higher Education Institutions: a Systematic Literature Review	To know the individual readiness to participate in a KM initiatives	Universities	Trust	Tested for internal consistency and convergent and discriminant validity	Apart from trust were rated higher than the variables that were associated with the relationship with colleagues.	Faculty feel whether their college ready to opt KM
Keshavarz et al., 2018	Knowledge management infrastructures and organizational intelligence in Iranian research centers	In two countrywide research centers, the goal of this article is to investigate the probable link between knowledge management infrastructures (KMI) and organizational intelligence (OI).	Research Centers	Knowledge Management Infrastructure	The study is structured as a descriptive survey. The research was done amongst 175 faculty members and staff members of the two research institutes using two questionnaire techniques relating to KMI and OI. The obtained data were then examined using structural equation modeling (SEM) techniques and partial least squares.	Considering the two research hypotheses, path analysis revealed a significant link between two variables with a 95% confidence level.	–
Ojo, 2016	Knowledge Management in Nigerian Universities: A Conceptual Model	To contribute to the development of a learning society	Nigerian Universities	innovation	To investigate the notion of knowledge management and its implementation in higher education institutions, a literature review was done.	knowledge management has the potential for improving performance within universities, the proposed model must be subjected to empirical validation for further amendments and improvements	–
Chawla and Saaxena, 2014	A Conceptual Model	The goal of this article is to look at the reliability and validity of Lawson’s (2003) knowledge management assessment instrument (KMAI), which consists of 24 components.	Manufacturing companies	Knowledge Application	A total of 225 research scientists and 225 faculty members from nine higher education institutions took part in the survey. The respondents were given a five-point Likert scale to answer on, ranging from strongly agree to strongly disagree. The data was then examined using the software programs SPSS 18.0 and AMOS 18.0. Prior to completing a confirmatory factor analysis, an exploratory factor analysis was performed.	The results suggest that job embeddedness is a stronger negative predictor of the participants’ turnover	–
Agarwal and Marouf, 2014	Initiating Knowledge Management in Colleges and Universities: A template	to emphasize the significance of knowledge management in colleges and institutions	Higher Education Institution in Kashmir	Gender	Mean, Standard Deviation and One way ANOVA test	There are three decades of KM research and case studies scattered across the literature. Furthermore, the majority of KM research and case studies are focused on for-profit businesses.	–
Masa’deh et al., 2017	The impact of knowledge management on job performance in higher education The case of the University of Jordan	to look at the influence of intellectual capital on innovation in Jordanian telecommunications enterprises, using knowledge management as a mediator	University of Jordan	knowledge management	In the suggested study model, a structural equation modeling technique based on AMOS 22.0 was employed to analyze causal links and test hypotheses between observable and latent components.	Since a result of the findings, a mediation model was confirmed, as intellectual capital had no direct influence on creativity. Intellectual capital has a considerable influence on knowledge management and creativity, according to the findings. Implications for philosophy and practice are examined in light of these findings.	–
Bureš et al., 2011	RETHINKING OF KNOWLEDGE	To Identify Knowledge Communities	Czech Republic Teaching Universities	Quality Control Mechanisms	Business section of the Proust database	Human resources, culture, leadership, technology, curriculum, and quality control mechanisms are the six key pillars of KM implementation, as are four knowledge processes – producing, storing, sharing, and applying knowledge.	–
Ivanovic and Milenkovski, 2019	Importance Of New Approaches In Education For Higher Education Institutions	To draw attention to the needs and expectations of modern society in terms of developing human resources with appropriate skills and competencies for the labor market.	Education	Structural Literature Review	Conceptual	The results show that south-eastern European nations have lower-than-average performance when compared to EU countries, but there is still room for improvement, particularly through the use of computer-based technology.	–

## Result and Discussion

According to the findings of several studies, top management knowledge value and knowledge-based rewards improve innovation speed and reliability. Although a knowledge-oriented culture helps to improve the quality of innovation, it has little effect on the speed of invention. Furthermore, the information sharing process acts as a mediator between all of these knowledge management enablers and the pace and efficiency of the invention. Knowledge management has an effect on business education due to the mediator of the educational course and the impact on the corporate environment, according to the findings. Transformational leaders, information sharing, and innovation all had a beneficial direct impact. Furthermore, information sharing was discovered to be a link between transformative leadership and creativity.

As a result, permitting barrier-free knowledge sharing in higher education would foster transformative leadership. Human resources, leadership, culture, curriculum, technology, and quality assurance mechanisms are the six key pillars of KM implementation, as are four knowledge processes: producing, sharing, storing, and applying knowledge. According to the findings, knowledge-based leadership has a direct and beneficial impact on organizational performance. Furthermore, the data show that knowledge management systems and innovation help to moderate the effect of knowledge-oriented leadership on organizational performance. The findings show that knowledge management characteristics differ across local and international businesses, as well as their influence across organizational levels. This suggests that, while KM methods are expected to enhance HEI innovations, discrepancies in their dimensions show the necessity for careful selection of appropriate dimensions in order to achieve desired outcomes. The study’s major goal was to examine the knowledge management strategies of private and public university professors.

This research helps to understand that countries are investing so much in their educational sectors to meet the demand which is fuelled by the ever-increasing populations of school-going children, youth and adults seeking education at all levels. With Artificial Intelligence (AI) entering all sectors, higher education is no-exception but the education sector traditionally has been characterized by a slow development of knowledge and deprived in smart systems of knowledge management. Implementation of Artificial Intelligence in Knowledge Management will enable speedy and efficient decision-making with better accuracy and quality. AI-based KM systems can provide much of the required information and support in managing interdisciplinary knowledge and progressing to bring together communities of educators and subject matter experts from other multidisciplinary areas including social sciences, psychology, management, law & regulations, medical, anthropology and many other fields of knowledge.

## Conclusion

One of the majorly deprived areas of education and research is “Multidisciplinary.” Most studies reveal that the research produced by experts in most cases in higher education has not been multidisciplinary. Even most of the major archives and indexing services are based on focused research that lacks multidisciplinary and interdisciplinary research. AI-based KM systems can provide much of the required information and support in managing interdisciplinary knowledge and progressing to bring together communities of educators and subject matter experts from other multidisciplinary areas including social sciences, psychology, management, law & regulations, medical, anthropology, and many other fields of knowledge. In terms of method, culture, technology, and assessment, it is found that there are no substantial differences in knowledge management techniques between professors at public and private colleges. In terms of leadership; however, there are substantial differences in KM approaches between public and private sector institutions. The findings reveal that private institutions have stronger knowledge management practices in terms of knowledge top management. The current study’s scope is confined to solely the KM practices of the surveyed colleges that agreed to participate. Furthermore, the current study looked at universities as a whole and did not take into account any variances in KM practices based on faculty, institution type, and so on. As a result, there is a lot of room for more investigation. Our understanding of KM practices at HEIs might be further expanded by including bigger sample size and more countries. A future study might also focus on individual KM practices in higher education, in order to identify common patterns and compare and contrast institutions from different nations.

## Author Contributions

DG: conceived and designed the analysis, collected the data, contributed data, performed the analysis, and wrote the paper. NL: verified the data collected, co-wrote the paper, and finalized the paper. All authors contributed to the article and approved the submitted version.

## Conflict of Interest

The authors declare that the research was conducted in the absence of any commercial or financial relationships that could be construed as a potential conflict of interest.

## Publisher’s Note

All claims expressed in this article are solely those of the authors and do not necessarily represent those of their affiliated organizations, or those of the publisher, the editors and the reviewers. Any product that may be evaluated in this article, or claim that may be made by its manufacturer, is not guaranteed or endorsed by the publisher.
